# Supplementary material for: Far‐red light promotes Botrytis cinerea disease development in tomato leaves via jasmonate‐dependent modulation of soluble sugars
Source: Plant Cell Environ. 2020 Sep 4;43(11):2769–81. doi: 10.1111/pce.13870 (PMC7693051; doi:10.1111/pce.13870)
Supplement: Supplementary file 1 — Appendix S1: Supporting information [file PCE-43-2769-s001.pdf]

Supporting information

Figure S1: Local FR illumination setup. The top leaflet of leaf n°3 was illuminated from the bottom (**A**) by using FR LEDs attached to flexible arms (**B**). Infrared pictures showing FR illumination of the center of the lamina (**C**).

Figure S2: *Botrytis cinerea* mycelial growth is enhanced by elevated soluble sugar concentrations *in planta*. Mycelium diameter growth measurement after three days on water-based agar media supplemented with either water (Control) WL-treated or WL+FR-treated leaf homogenate (WL and WL+FR, respectively). Data represent mean ± SEM and asterisk represent significant difference (Student's t-test, $p < 0.05$, $n = 10$).

Figure S3: Local FR enrichment triggers petiole elongation. Petiole elongation measurements of the third and fourth leaf of three-week-old tomato plants after five days of local FR illumination on the top leaflet of the third leaf. Measurements were performed at the start and after five days of WL or local FR treatment light treatment (grey and red bars, respectively). Data represent three replicates merged together (n = 6 - 8 plants). Plotted data correspond to mean ± SEM. Different letters represent significant differences (ANOVA Tukey's post-hoc test, $p < 0,05$).

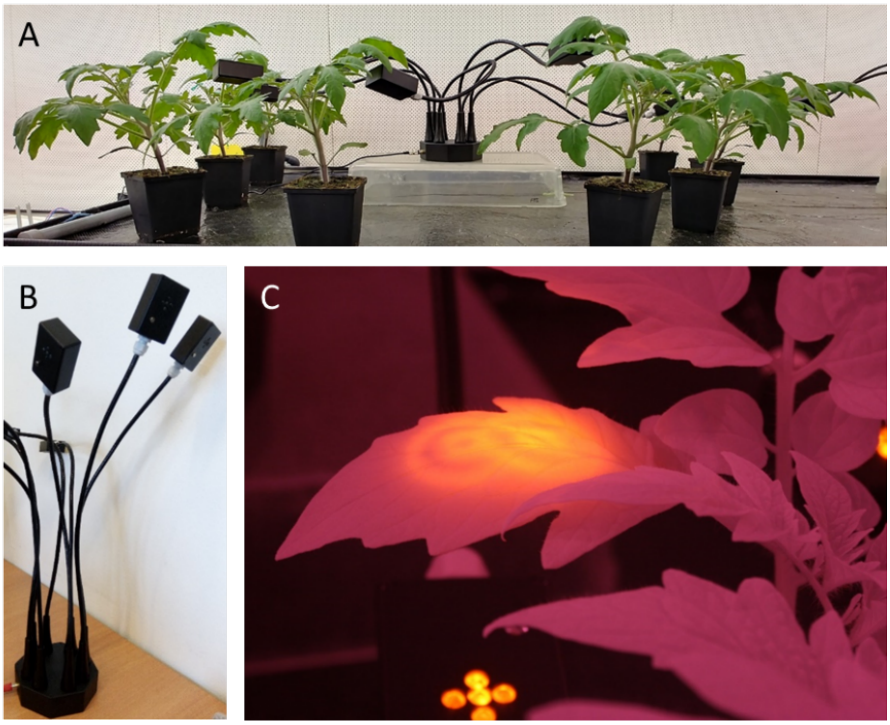
A
B
C

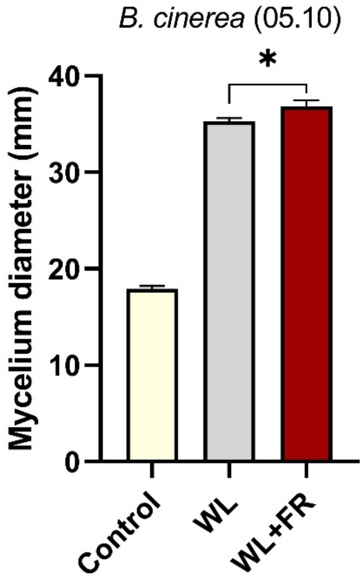

B. cinerea (05.10)
*
40
30
20
10
0
Mycelium diameter (mm)
Control
WL
WL+FR

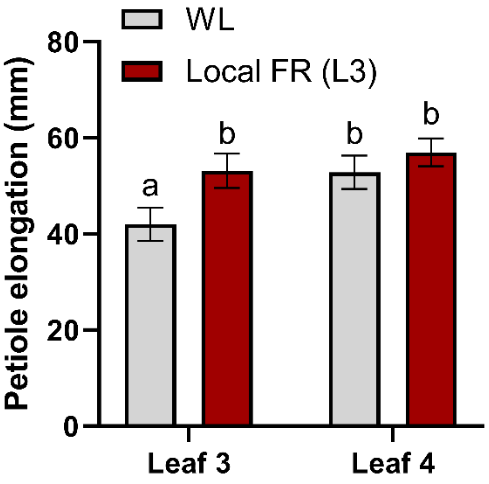
WL
Local FR (L3)
Petiole elongation (mm)
80
60
40
20
0
a
b
b
b
Leaf 3
Leaf 4
